# Predictive value of routine ultrasound indicators in the second trimester for gestational diabetes mellitus: a retrospective cohort study

**DOI:** 10.3389/fendo.2026.1873073

**Published:** 2026-07-02

**Authors:** Chaoran Liu, Hao Su, Xiaohui Ma, Zhihui Liu

**Affiliations:** 1 Affiliated Baotou Central Hospital of Baotou Medical College, Baotou, China; 2 Department of Anesthesiology, Baotou Central Hospital, Baotou, China; 3 Department of Endocrinology, Hohhot First Hospital, Hohhot, China

**Keywords:** gestational diabetes mellitus, predictive value, retrospective cohort study, second trimester, ultrasound parameters

## Abstract

**Background:**

Gestational diabetes mellitus (GDM) is associated with adverse maternal and offspring outcomes, yet the predictive value of routine second-trimester ultrasound parameters remains unclear. This study aimed to systematically evaluate the ability of common fetal ultrasound indices measured at 19–24 weeks to predict GDM.

**Methods:**

This retrospective cohort study initially enrolled 254 singleton pregnancies. After exclusions, 241 women (117 with GDM, 124 controls) were analyzed. Nine ultrasound parameters (biparietal diameter, head circumference, abdominal circumference, femur length, amniotic fluid depth, fetal heart rate, humeral length, placental thickness, and estimated fetal weight) were converted to gestational age-adjusted Z-scores. Individual and combined predictive performances were assessed using ROC curves, multivariable logistic regression, calibration curves, and decision curve analysis (DCA).

**Results:**

Among single ultrasound indices, only amniotic fluid depth showed statistical significance, albeit with a modest AUC of 0.588 (95% CI: 0.517–0.658, P = 0.017). After adjusting for pre-pregnancy BMI, amniotic fluid depth remained independently associated with GDM but in an inverse direction (OR = 0.754, 95% CI: 0.575–0.989, P = 0.041). The combined model (pre-pregnancy BMI plus six ultrasound Z-scores) achieved an AUC of 0.692 (95% CI: 0.625–0.758, P< 0.001), with good calibration (Hosmer-Lemeshow P = 0.339). DCA showed a modest positive net benefit within the threshold probability range of 0.1–0.3.

**Conclusion:**

Routine fetal ultrasound parameters at 19–24 weeks have very limited predictive value for GDM. The combined model including pre-pregnancy BMI failed to reach the clinically useful AUC of 0.70. These routine indices are not recommended for GDM screening before 24 weeks. Current practice of OGTT at 24–28 weeks should remain the standard. Future research should focus on later gestational windows or more specific sonographic biomarkers such as fetal pancreatic circumference and liver volume.

## 1 Introduction

Gestational diabetes mellitus (GDM) is defined as glucose intolerance first recognized during pregnancy (1). With the rising prevalence of obesity and the increasing number of advanced-age mothers following the adjustment of China’s fertility policy, GDM has become one of the most common pregnancy complications nationwide (2). Beyond the acute perinatal risks, women with GDM face a significantly higher long-term risk of type 2 diabetes, and their offspring are more prone to obesity and metabolic disorders (3). Notably, a history of GDM increases the subsequent risk of cardiovascular disease by 1.7-fold and has been recognized by the American Heart Association as a sex-specific risk factor for women (4–6). These observations underscore the need for early identification of at-risk pregnancies.

Current routine screening for GDM relies on the oral glucose tolerance test (OGTT) performed at 24–28 weeks of gestation (7). Although this approach is well-established, its late timing limits the window for early lifestyle or dietary interventions that might ameliorate maternal and neonatal outcomes (8). Moreover, the OGTT requires multiple blood draws and can be poorly tolerated, occasionally leading to unreliable results. Therefore, a simpler, earlier, and less burdensome prediction tool would be of clinical value.

Fetal ultrasound, being non-invasive, repeatable, and already integrated into routine antenatal care, has been explored for early GDM prediction. In the late third trimester, several studies have shown that increased fetal abdominal circumference and amniotic fluid index are significantly associated with GDM (9). However, evidence in the mid-trimester (20–24 weeks) remains conflicting. Some reports suggest that fetal head circumference or femur length above the 90th percentile at 20 weeks may increase GDM risk (10, 11), while others fail to replicate such associations (12), This inconsistency may stem from differences in study populations, GDM diagnostic criteria, and sample sizes (13, 14).Importantly, most previous work has focused on selected individual parameters, and a systematic evaluation of the routine ultrasound measurements (head circumference, abdominal circumference, biparietal diameter, femur length, amniotic fluid depth, fetal heart rate, humeral length, placental thickness, and estimated fetal weight) that are universally reported in standard second-trimester scans is still lacking (15, 16).

In this study, we therefore aimed to systematically assess the predictive value of these nine routine fetal ultrasound parameters, both individually and in combination, for GDM using real-world data from 19–24 weeks of gestation. We further examined whether adding pre-pregnancy body mass index could improve the predictive performance. By providing a comprehensive evaluation of these common measures, we hope to clarify their clinical utility (or lack thereof) in early GDM risk stratification—negative findings herein are valuable for avoiding futile screening and steering research toward better markers.

## 2 Materials and methods

### 2.1 Study participants

This is a case-enriched retrospective cohort study. During the study period (January 2023 to February 2026), a total of 3,225 singleton deliveries were recorded at our institution, of which 323 (10.0%) were diagnosed with gestational diabetes mellitus (GDM) using the Carpenter-Coustan criteria. From this source population, we initially enrolled 254 singleton pregnant women who underwent routine prenatal ultrasound and subsequently delivered at our hospital. Based on the 24–28 week oral glucose tolerance test (OGTT) results, 130 were preliminarily diagnosed with GDM and 124 served as normoglycemic controls.

Inclusion criteria were: (1) naturally conceived singleton pregnancy; (2) accurate gestational age confirmed by last menstrual period; (3) absence of pregestational diabetes, fetal structural anomalies, or major maternal comorbidities. Exclusion criteria were: (1) pregestational diabetes or first-trimester fasting plasma glucose ≥7.0 mmol/L; (2) incomplete OGTT data; (3) severe placental disorders or fetal malformations.

**Figure 1:**
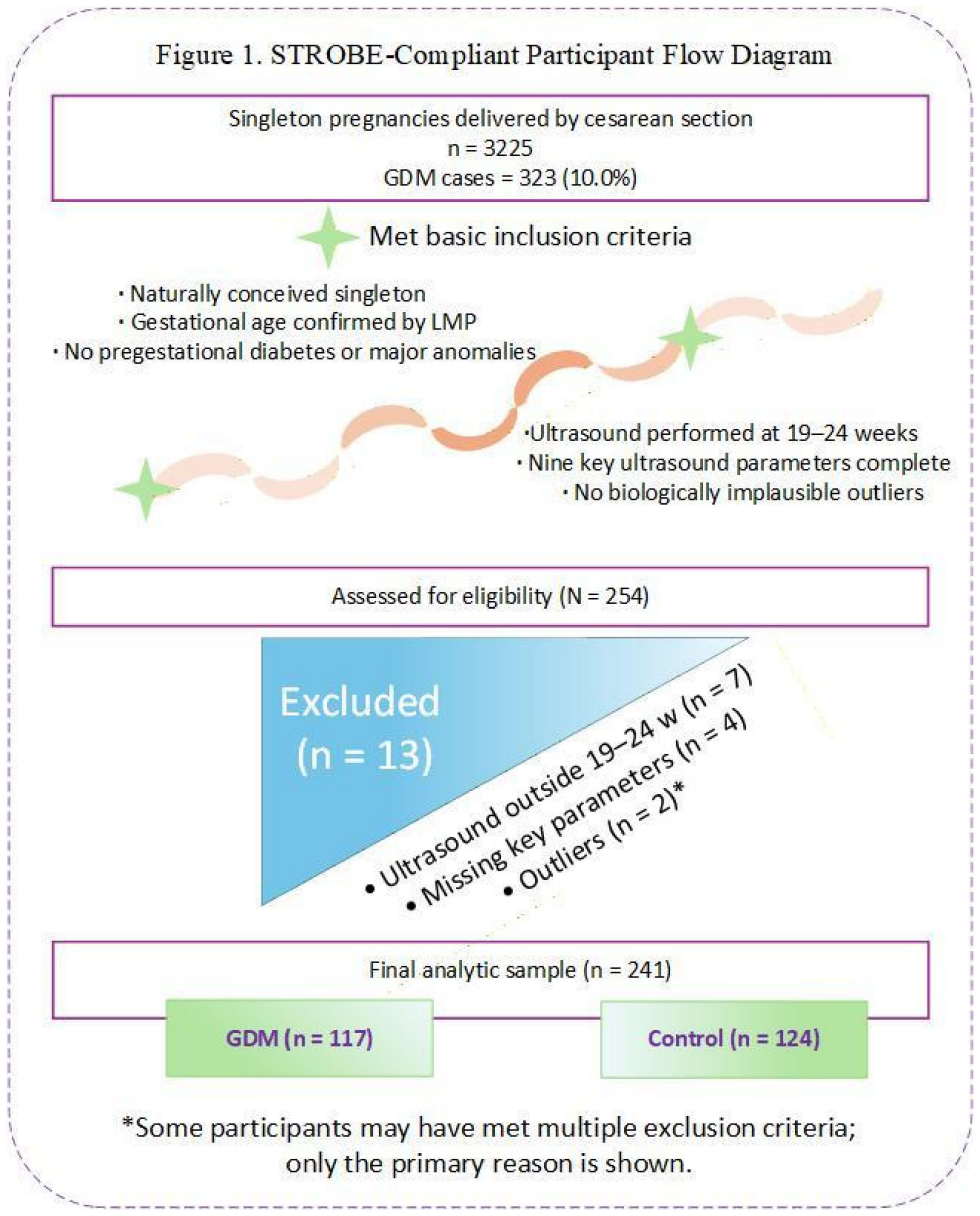
Flowchart illustrating participant selection for a study on gestational diabetes, starting from the total of 3,225 singleton deliveries at our institution during the study period, applying inclusion and exclusion criteria, excluding 7 cases with ultrasound outside 19–24 weeks, 4 cases with missing key ultrasound parameters, and 2 cases with biologically implausible measurement outliers, resulting in a final analytic sample of 117 GDM cases and 124 controls.

After exclusions, 241 participants were included in the final analysis. Reasons for exclusion were: ultrasound examination outside the 19–24 week window (n=7); complete absence of key ultrasound parameters due to not undergoing the scheduled scan that month (n=4); and biologically implausible measurement outliers (n=2). Outliers were defined as any raw measurement that, when converted to standard clinical units (cm or mm), fell beyond ±4 standard deviations of the institutional reference mean or violated anatomical plausibility (e.g., head circumference smaller than biparietal diameter). Examples included femur length equivalent to<18 weeks or >30 weeks of gestation. The final analysis comprised 117 women in the GDM group and 124 in the control group. The participant selection process is illustrated in **Figure 1**.

The four cases with missing ultrasound parameters were classified as missing completely at random (MCAR), because the absence was due to logistical reasons (the participants did not undergo the scheduled scan that month) rather than any clinical characteristics or GDM status. Given the low missing proportion (4/254, 1.6%), complete-case analysis was performed without imputation.

### 2.2 GDM diagnostic criteria

A two-step screening approach was adopted. All pregnant women underwent a 50g glucose challenge test (GCT) at 24–28 weeks of gestation. Those with a 1-hour plasma glucose level ≥7.8 mmol/L proceeded to a 100g oral glucose tolerance test (OGTT). According to the Carpenter-Coustan criteria, GDM was diagnosed if any two of the following plasma glucose thresholds were met or exceeded: fasting 5.3 mmol/L, 1-hour 10.0 mmol/L, 2-hour 8.6 mmol/L, or 3-hour 7.8 mmol/L.

### 2.3 Ultrasound indicator acquisition

Ultrasound examinations were performed using GE Voluson E8/E10 ultrasound systems by three operators with at least five years of experience in obstetric ultrasound. During routine fetal systematic ultrasound examination, the following parameters were measured at the same time point: biparietal diameter (BPD), head circumference (HC), abdominal circumference (AC), femur length (FL), maximum amniotic fluid depth (AFD), fetal heart rate (FHR), humerus length (HL), placental thickness (PT), and estimated fetal weight (EFW). All measurements were taken as the average of three repeated values.

### 2.4 Data standardization

To eliminate the effect of gestational age on the measurements, linear regression models of each ultrasound indicator against gestational age were established using normal pregnant women without GDM or other complications (n=124) as the reference. Taking head circumference as an example, the regression equation was HC_pred = a × GA + b, where GA is gestational age in weeks. The R^2^ values of all regression models ranged from 0.52 to 0.68, indicating that gestational age explained more than 50% of the variance in each indicator. The predicted value for each fetus was calculated based on the regression model, and the Z-score was then computed as:


$$Z=\frac{\left(\text{Measured value}-\text{Predicted value}\right)}{\text{Residual standard deviation}}$$

The residual standard deviation was defined as the sample standard deviation of the residuals in the normal control group. The Shapiro–Wilk test confirmed that the residuals of each indicator followed a normal distribution (P > 0.05), and no obvious heteroscedasticity was observed in the residual plots. Although the linear correlation between fetal heart rate and gestational age was weak (R^2^ = 0.09), the same method was still applied to calculate its Z-score to maintain consistency across all indicators.

### 2.5 Sample size estimation

This was a retrospective cohort study, and the sample size was based on convenience sampling (all eligible cases meeting the inclusion and exclusion criteria from January 2023 to February 2026). The final analysis included 117 GDM events, with seven independent variables (including pre-pregnancy BMI and six ultrasound Z-scores), yielding an events-per-variable (EPV) ratio of approximately 12.7, which meets the empirical rule minimum of 10:1 but is slightly below the more stringent criterion of 15:1. Based on this, a *post hoc* power analysis was performed. Assuming a significance level of α = 0.05 and a moderate effect size (OR ≈ 0.75), the current sample size provided approximately 78% power to detect this effect. For weaker effects (e.g., OR ≥ 0.85), the power was below 60%. Therefore, this study has basic power to detect moderate or larger effects, but interpretation of weaker effects should be cautious.

### 2.6 Statistical analysis

Continuous variables were first tested for normality using the Shapiro-Wilk test. Normally distributed data are presented as mean ± standard deviation (SD) and were compared using the independent-samples t-test; non-normally distributed data are presented as median (interquartile range) and were compared using the Mann-Whitney U test. Categorical variables are presented as number (percentage) and were compared using the chi-square test or Fisher’s exact test, as appropriate.

For each ultrasound Z-score, receiver operating characteristic (ROC) curves were constructed, and the area under the curve (AUC) with its 95% confidence interval (CI) was calculated. The DeLong test was used to determine whether the AUC was significantly greater than 0.5. An AUC ≥ 0.70 was considered clinically acceptable.

Binary logistic regression (Enter method) was performed with GDM status (1 = yes, 0 = no) as the dependent variable and pre-pregnancy BMI together with six ultrasound Z-scores (head circumference, abdominal circumference, biparietal diameter, femur length, amniotic fluid depth, and fetal heart rate) as independent variables. Missing rates for these variables were<5%. The predicted probability (PRE-1) generated by the regression model was then used to construct a combined ROC curve.

Calibration of the combined model was assessed using the Hosmer-Lemeshow goodness-of-fit test and visualized with a calibration curve (predicted probability versus observed proportion). Clinical utility was evaluated using decision curve analysis (DCA), comparing the net benefit of the prediction model with the “treat all” and “treat none” strategies across a range of threshold probabilities (17).

HbA1c was not included in the multivariable logistic regression model because it is not a routine screening test in early pregnancy at our institution. It is measured only when clinically indicated (e.g., suspected pregestational diabetes), resulting in available data for only 38 of 241 participants (15.8%). Including HbA1c would have reduced the sample size by 84% and introduced selective bias due to non-random missingness (missing not at random, MNAR). Therefore, pre-pregnancy BMI was used as the primary covariate, which is a consistently measured and well-established risk factor for GDM.

All statistical tests were two-sided, and a P value< 0.05 was considered statistically significant. Data analysis was performed using SPSS version 26.0 (IBM Corp., Armonk, NY, USA) and GraphPad Prism 9.5 (GraphPad Software, San Diego, CA, USA).

## 3 Results

### 3.1 Baseline characteristics

A total of 241 participants were included in the final analysis: 117 with GDM and 124 normoglycemic controls. The two groups were comparable in terms of age, number of pregnancies, and parity (all P > 0.05). As expected, the GDM group had significantly higher pre-pregnancy BMI (25.19 ± 4.18 kg/m^2^ vs. 22.90 ± 3.63 kg/m^2^, P< 0.001). Detailed comparisons are presented in **Table 1**.

**Table 1:** Comparison of baseline clinical characteristics between the GDM group and the control group.

Variable	GDM group (n=117)	Control group (n=124)	P
Age (years)	32.32 ± 4.08	31.90 ± 5.02	0.535
Pre-pregnancy BMI (kg/m^2^)	25.19 ± 4.18	22.90 ± 3.63	<0.001
US GA (wk), median (IQR)	20.1 (19.4–20.9)	20.0 (19.3–20.8)	
OGTT GA (wk), median (IQR)	25.2 (24.6–25.9)	–	–
Gravidity	1.50(0.90-2.30)	1.80(1.10-2.70)	0.068
Parity	0.50(0.20-1.00)	0.60(0.30-1.10)	0.440

*Continuous variables were tested for normality by Shapiro-Wilk test. Age and pre-pregnancy BMI were normally distributed, presented as mean ± SD (independent samples t-test). Gravidity and parity were non-normally distributed, presented as median (IQR) (Mann-Whitney U test). *P< 0.05 was considered statistically significant. OGTT was performed only in women with a positive GDM screening test; data not available for controls.*.

To assess the potential impact of excluding outliers and missing data, two sensitivity analyses were conducted. First, the two outlier measurements were winsorized at ±4 SD and re-analyzed; the direction and significance of the association between amniotic fluid depth Z-score and GDM remained unchanged (OR changed by<0.01, P value variation<0.01). Second, under extreme-case scenarios assigning the four missing cases to either GDM or control groups, the AUC of the combined model varied by no more than ±0.02. These results suggest that the exclusions did not substantially bias the main conclusions.

### 3.2 Neonatal outcomes

No significant differences were observed between the two groups in gestational age at delivery, birth weight, 1-minute Apgar score, or stillbirth rate. A summary of neonatal outcomes is provided in **Table 2**.

**Table 2:** Comparison of neonatal outcomes between the two groups.

Variable	GDM group (n=117)	Control group (n=124)		P
Gestational age at delivery (weeks)	39.23 ± 1.42		38.83 ± 1.75	0.142
Birth weight (g)	3271.69 ± 575.00		3357.60 ± 537.11	0.316
1-minute Apgar score	9.67 ± 0.58		9.73 ± 0.53	0.488
Stillbirth, n (%)*	1 (1.1)		0 (0)	0.320

Gestational age at delivery, birth weight, and 1-minute Apgar score were normally distributed and are presented as mean ± standard deviation; between-group comparisons were performed using independent samples t-test. Stillbirth is presented as number (%), and between-group comparison was performed using Fisher’s exact test (due to expected frequency<5). A P value< 0.05 was considered statistically significant.

### 3.3 Individual predictive performance of ultrasound parameters

**Figure 2:**
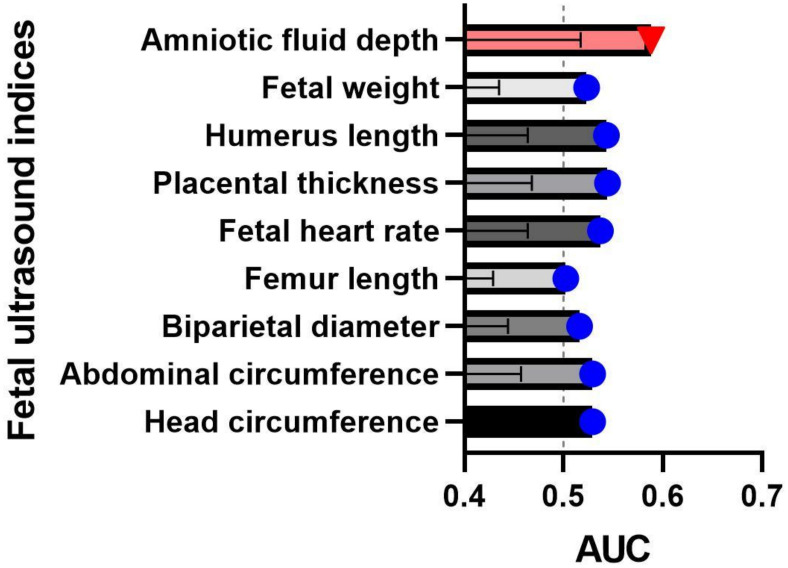
Each point represents the AUC of a single ultrasound parameter, with error bars indicating 95% confidence intervals.

ROC curve analyses for each ultrasound Z-score are summarized in **Table 3**. Only amniotic fluid depth Z-score showed a statistically significant, albeit modest, AUC of 0.588 (95% CI: 0.517–0.658, P = 0.017). For all other eight parameters, AUC values were below 0.55, and their 95% confidence intervals crossed 0.5, indicating no reliable discriminative ability (**Figure 2**). Detailed results are shown in **Table 3**.

**Table 3:** Multivariate logistic regression analysis of the relationship between ultrasound indicators and GDM.

Variable	B	SE	Wald χ^2^		P	OR	95% CI
BMI (kg/m^2^)	0.153	0.038	16.378	<0.001		1.165	1.082–1.254
Head circumference	-0.093	0.146	0.408	0.523		0.911	0.684–1.213
Biparietal diameter	0.042	0.130	0.102	0.749		1.042	0.808–1.344
Abdominal circumference	-0.089	0.148	0.363	0.547		0.914	0.684–1.223
Femur length	0.000	0.136	0.000	0.995		0.999	0.765–1.304
**Amniotic fluid depth**	**-0.282**	**0.138**	**4.176**	**0.041**		**0.754**	**0.575–0.989**
Fetal heart rate	0.130	0.145	0.804	0.370		1.138	0.858–1.511

All ultrasound indicators were entered into the model as Z-scores (using the normal control group as a reference to eliminate the effect of gestational age). GDM was coded as 1 = yes, 0 = no. Bold values indicate the only statistically significant positive finding (P < 0.05).

Given that the negative association between amniotic fluid depth and GDM appeared inconsistent with objective data, we re-examined the amniotic fluid measurements. Univariate analysis further compared the raw Z-scores of amniotic fluid depth between the two groups. The mean amniotic fluid depth Z-score was significantly lower in the GDM group than in the control group (–0.287 ± 0.150 vs. 0.000 ± 0.148; mean difference –0.287, 95% CI –0.539 to –0.035; independent samples t-test, P = 0.026). This unadjusted negative association was consistent with the subsequent multivariable logistic regression, in which the amniotic fluid depth Z-score remained independently and negatively associated with GDM after adjustment for pre-pregnancy BMI (OR = 0.754, 95% CI 0.575–0.989, P = 0.041).

### 3.4 Multivariable logistic regression and combined prediction model

Pre-pregnancy BMI and six ultrasound Z-scores (head circumference, abdominal circumference, biparietal diameter, femur length, amniotic fluid depth (18), and fetal heart rate) were entered into a binary logistic regression model using the Enter method. As shown in **Table 4**, pre-pregnancy BMI was significantly and positively associated with GDM (OR = 1.165, 95% CI: 1.082–1.254, P< 0.001). After adjusting for pre-pregnancy BMI, only amniotic fluid depth Z-score remained independently associated with GDM, showing a negative association (OR = 0.754, 95% CI: 0.575–0.989, P = 0.041). None of the other ultrasound Z-scores reached statistical significance.

**Table 4:** List of abbreviations.

Column name	Full name
B	Regression coefficient
SE	Standard Error
Wald χ^2^	Wald Chi-Square statis
P	P-value
OR	Odds Ratio
95% CI	95% Confidence Interval
US GA (wk), median (IQR)	Gestational age at ultrasound (weeks), median (IQR)
OGTT GA (wk), median (IQR)	Gestational age at OGTT (weeks), median (IQR)

**Figure 3:**
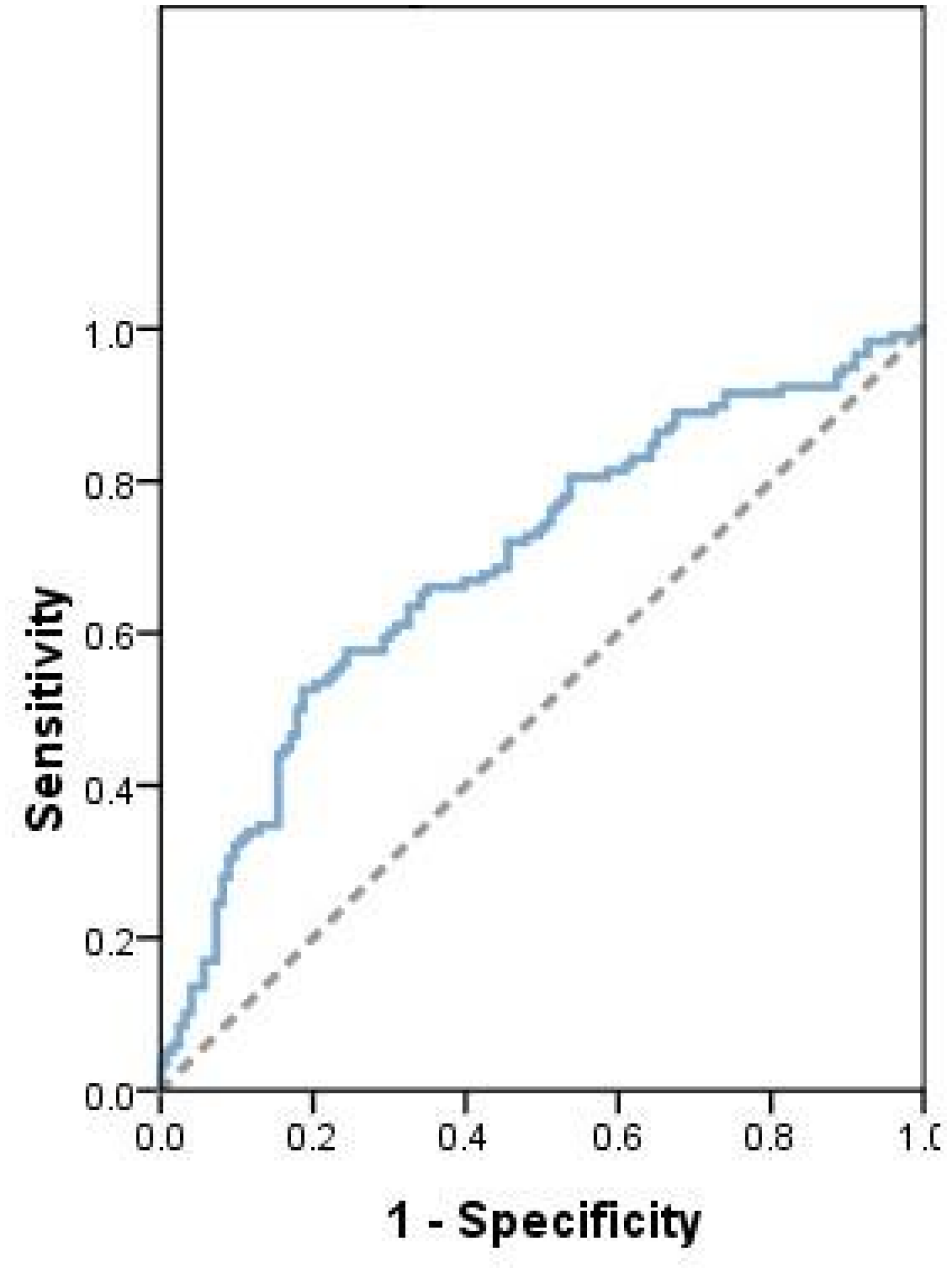
The combined model includes pre‑pregnancy BMI and Z‑scores of head circumference, abdominal circumference, biparietal diameter, femur length, amniotic fluid depth, and fetal heart rate. AUC = 0.692 (95% CI: 0.625–0.758).

The predicted probability (PRE-1) derived from the regression model was used to construct an ROC curve for the combined model. The combined model achieved an AUC of 0.692 (95% CI: 0.625–0.758, P< 0.001; **Figure 3**).

### 3.5 Model calibration and decision curve analysis

**Figure 4:**
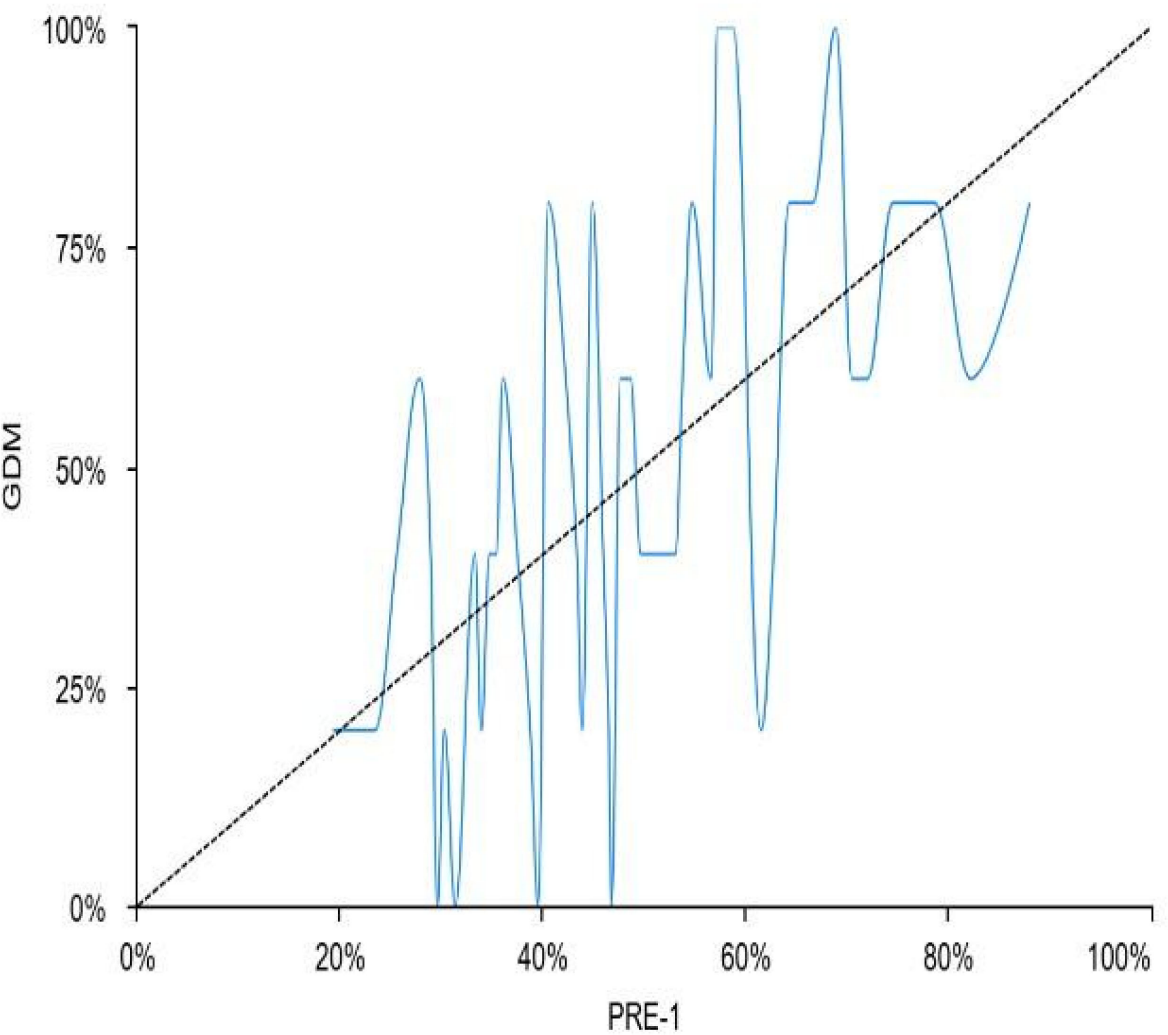
The diagonal line represents perfect calibration. Hosmer‑Lemeshow test P = 0.339.

The Hosmer-Lemeshow test yielded a P value of 0.339, indicating no significant lack of fit. The calibration curve showed good agreement between predicted probabilities and observed GDM rates (**Figure 4**).

**Figure 5:**
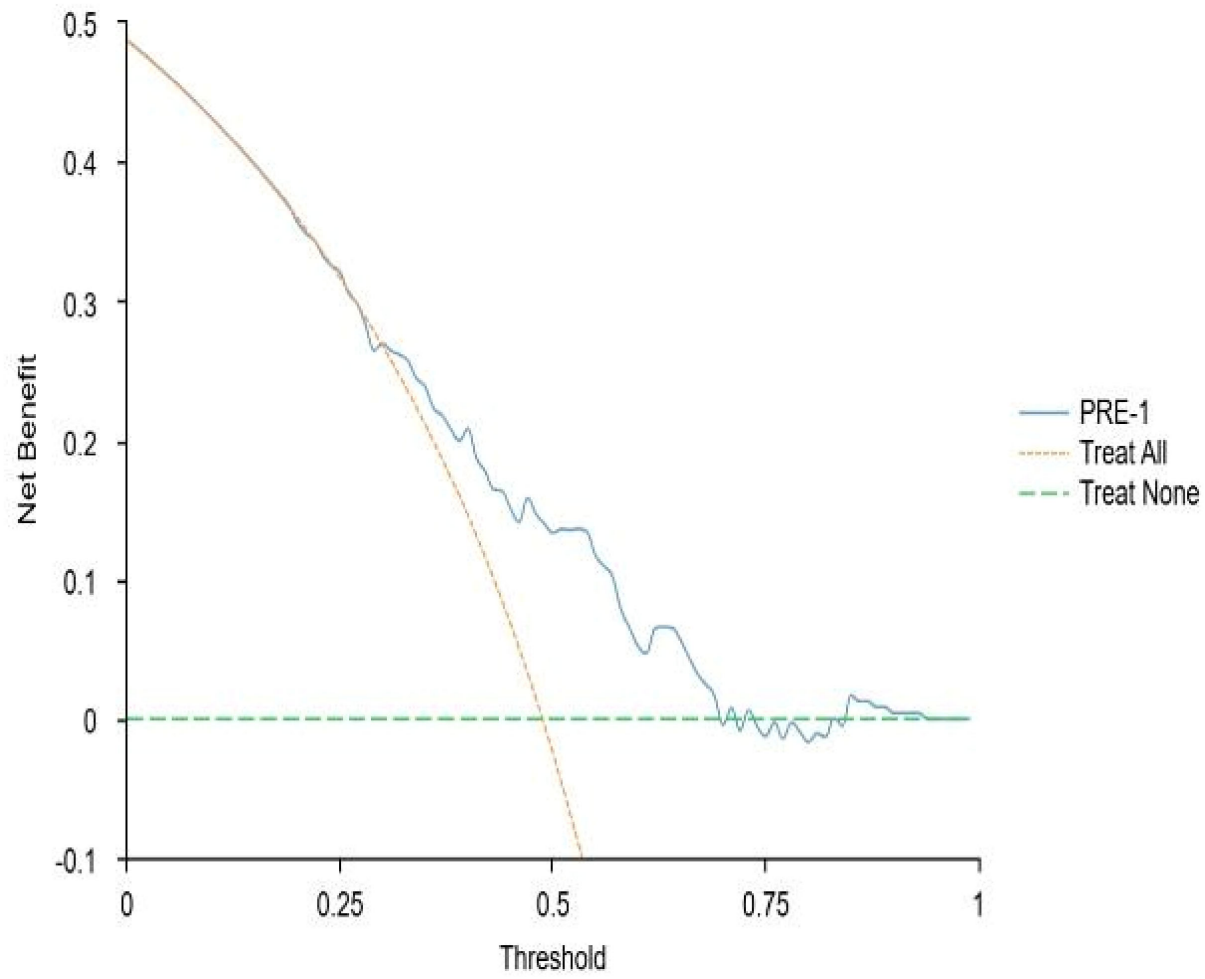
Decision curve analysis showed a modest positive net benefit only when clinicians were willing to intervene at a very low risk threshold (0.1–0.3), which might correspond to ‘ruling out’ low-risk women to reduce unnecessary OGTTs rather than ‘ruling in’ GDM. However, given the AUC < 0.70, this potential utility remains theoretical and requires external validation before any clinical application.

Decision curve analysis showed a modest positive net benefit only when clinicians were willing to intervene at a very low risk threshold (0.1–0.3), which might correspond to ‘ruling out’ low-risk women to reduce unnecessary OGTTs rather than ‘ruling in’ GDM (19). However, given the AUC< 0.70, this potential utility remains theoretical and requires external validation before any clinical application. (**Figure 5**).

### 3.6 Sensitivity analysis for temporal sequence

To assess whether the temporal order between ultrasound (19–24 weeks) and OGTT (24–28 weeks) affected the results, we performed a sensitivity analysis by excluding the 11 GDM cases (9.4% of the GDM group) whose OGTT was performed before ultrasound (OGTT gestational age<19 weeks). Univariable logistic regression for amniotic fluid depth Z-score — the only ultrasound parameter that was statistically significant in the primary analysis — was repeated in the remaining 106 GDM cases and 124 controls. The OR changed minimally from 0.754 (95% CI: 0.575–0.989, P = 0.041) to 0.758 (95% CI: 0.574–0.998, P = 0.048), and the AUC changed from 0.588 to 0.584 (95% CI: 0.512–0.656). These results indicate that the reversed temporal sequence did not materially bias the main conclusions.

## 4 Discussion

### 4.1 Main findings and interpretation

In this retrospective cohort study of 241 singleton pregnancies, we systematically evaluated the predictive value of nine routine fetal ultrasound parameters measured at 19–24 weeks of gestation for GDM. Specifically, three findings warrant discussion. First, isolated amniotic fluid depth showed only a weak and inverse association with GDM (OR = 0.754), which contradicts the expected positive relationship seen in later pregnancy. This suggests that the pathophysiology of amniotic fluid regulation in GDM—namely fetal osmotic diuresis secondary to hyperglycemia—may not yet be operative at 19–24 weeks, or may be counteracted by other factors such as early glycemic control. Second, no other single biometric parameter (head circumference, abdominal circumference, femur length, etc.) reached statistical significance, reinforcing that routine fetal growth metrics lack the temporal and organ-specific sensitivity needed for early GDM detection. Third, even a combined model incorporating pre-pregnancy BMI and six ultrasound Z-scores failed to reach the clinically accepted AUC threshold of 0.70 (AUC = 0.692), although DCA suggested a marginal net benefit at very low risk thresholds.

Taken together, these findings shift the clinical question from “which ultrasound parameter predicts GDM?” to “why do routine second-trimester parameters fail to predict GDM?” The most plausible explanation is that fetal overgrowth requires sufficient duration and intensity of hyperglycemic exposure, typically emerging after 28 weeks, and that routine biometry lacks the metabolic specificity to detect early GDM-related changes. By contrast, emerging markers such as fetal pancreatic circumference or liver volume may offer earlier windows because they reflect direct metabolic stress rather than gross anthropometry.

Consequently, our negative results should not be seen as a failure but as actionable evidence: they discourage the use of routine second-trimester ultrasound for GDM screening, reinforce the current 24–28 week OGTT window, and direct future research toward more specific fetal soft-tissue or metabolic biomarkers measured at later gestational ages.

### 4.2 Why routine second-trimester ultrasound parameters fail to predict GDM

GDM is characterized by maternal hyperglycemia that leads to fetal overgrowth, but the effect is time-dependent. Fetal growth acceleration typically becomes detectable only after sufficient exposure to elevated glucose levels, which often occurs in the late second or third trimester. Our measurement window at 19–24 weeks may be too early to capture these changes. Indeed, studies reporting significant associations between ultrasound parameters and GDM have mostly focused on the late third trimester (e.g., fetal abdominal circumference at 28–32 weeks) or used specialized measures such as fetal pancreatic circumference and liver volume, which show measurable alterations as early as 20–25 weeks (20).

The lack of predictive ability for routine head, abdominal, and limb measurements in our study aligns with the notion that these non-specific growth metrics require a longer duration of hyperglycemic exposure to diverge between GDM and normal pregnancies. Therefore, our negative results are not contradictory to existing literature but rather complement it by highlighting the temporal and organ-specific limitations of routine biometry.

### 4.3 The unexpected inverse association of amniotic fluid depth

In our multivariable analysis, amniotic fluid depth Z-score was negatively associated with GDM (OR = 0.754), which seems to contradict the common clinical impression that GDM is associated with polyhydramnios. Several explanations merit consideration. First, the well-documented increase in amniotic fluid volume in GDM is primarily attributed to fetal osmotic diuresis secondary to hyperglycemia, but this mechanism requires sufficient fetal glomerular filtration and urine output, which become significant only after approximately 28 weeks (21). Our measurement at 19–24 weeks may have missed this effect. Second, the glycemic control in our GDM group was relatively good, and their average birth weight was even slightly lower than that of controls. Tight glycemic management might have attenuated the expected hyperglycemia-induced polyhydramnios. Third, the negative association could be a chance finding or reflect residual confounding, such as an unmeasured protective factor. Given the retrospective nature of our study, causal inference is limited, and this unexpected finding should be interpreted cautiously until confirmed by larger prospective cohorts.

### 4.4 The modest net benefit of the combined model: beyond AUC

The combined model yielded an AUC of 0.692, which is below the conventional diagnostic threshold of 0.70. Nevertheless, decision curve analysis demonstrated that within the threshold probability range of 0.1–0.3, the model provided a positive net benefit compared with both extreme strategies. This observation underscores an important distinction: AUC reflects overall discriminative ability across all thresholds, whereas DCA evaluates clinical utility at specific decision thresholds. In the context of GDM screening, clinicians may accept a lower threshold when the aim is to rule out low-risk women from unnecessary OGTTs rather than to confirm a diagnosis. Under such circumstances, even a modestly performing model could have practical value as a triage tool. That said, given the AUC< 0.70, we do not advocate using this model as a standalone screening test; its potential role, if any, should be limited to supporting shared decision-making prior to routine OGTT.

### 4.5 Comparison with emerging sonographic markers

Recent studies have reported promising early predictors of GDM, including fetal pancreatic circumference (15), liver volume (22), and visceral adipose tissue depth (23), measurable as early as 20–25 weeks. In contrast, our study found no predictive value for routine biometry. This contrast supports a shift in research focus: rather than continuing to refine conventional head, abdominal, and limb measurements, future efforts should prioritize more specific, organ-based markers that reflect the direct metabolic impact of hyperglycemia on fetal soft tissues and endocrine organs. Our negative findings thus provide a useful benchmark and underscore the need for future prospective studies using these novel markers.

### 4.6 Relationship between second-trimester ultrasound findings and neonatal outcomes

While our study observed no significant differences between GDM and control groups in basic neonatal outcomes (gestational age, birth weight, Apgar score, stillbirth), this does not negate the broader impact of second-trimester ultrasound on infant health. Ultrasound findings such as fetal growth restriction, oligo/polyhydramnios, or structural anomalies often trigger intensified surveillance, iatrogenic preterm delivery, or changes in intrapartum management, all of which can influence neonatal morbidity. Moreover, even normal ultrasound results may affect maternal stress and subsequent health behaviors. More subtle outcomes, such as neonatal antibiotic use (24), represent an important frontier for linking second-trimester biomarkers to infant health, but were not available in our retrospective cohort. Future prospective studies should incorporate these endpoints to fully understand the clinical impact of second-trimester ultrasound.

## 5 Limitations

Several limitations should be acknowledged. First, the retrospective design is subject to selection bias, and the sample size was based on convenience rather than *a priori* power calculation. Post-hoc power analysis indicated approximately 78% power to detect an OR of 0.75, but insufficient power for weaker effects. Second, some parameters (e.g., placental thickness, humeral length, estimated fetal weight) had relatively high missing rates and could not be included in the combined model, potentially underestimating the predictive performance of a multi-parameter approach. Third, the number of GDM events (117) restricted the number of covariates we could include, and external validation in an independent cohort is lacking. Fourth, the unexpected inverse association of amniotic fluid depth requires confirmation in larger prospective studies with standardized measurement protocols. Fifth, HbA1c was not included due to low availability (15.8% of participants) and non-random missingness (missing not at random, MNAR).Its inclusion would have caused substantial sample loss and selection bias. This limitation should be addressed in future studies with routine early-pregnancy HbA1c measurement. Finally, our findings may not generalize to populations with different ethnic backgrounds or GDM diagnostic criteria.

## 6 Conclusions

Routine fetal ultrasound parameters measured at 19–24 weeks of gestation have no independent predictive value for GDM, except for a weak inverse association with amniotic fluid depth. Even after adding pre-pregnancy BMI, the combined model achieved an AUC of only 0.69, which falls short of the clinically acceptable threshold (≥0.70). Therefore, we do not recommend using these routine ultrasound indices to screen for GDM before 24 weeks of gestation. Our negative findings indirectly support the current practice of OGTT screening at 24–28 weeks. Future research should explore later gestational windows or more specific sonographic biomarkers such as fetal pancreatic circumference and liver volume to enable truly early warning of GDM.

## Data Availability

The original contributions presented in the study are included in the article/supplementary material. Further inquiries can be directed to the corresponding author.
